# Structure-Function Relationships of the Neisserial EptA Enzyme Responsible for Phosphoethanolamine Decoration of Lipid A: Rationale for Drug Targeting

**DOI:** 10.3389/fmicb.2018.01922

**Published:** 2018-08-21

**Authors:** Charlene M. Kahler, K. L. Nawrocki, A. Anandan, Alice Vrielink, William M. Shafer

**Affiliations:** ^1^The Marshall Center for Infectious Diseases Research and Training, School of Biomedical Sciences, University of Western Australia, Crawley, WA, Australia; ^2^Perth Children’s Hospital, Telethon Kids Institute, Subiaco, WA, Australia; ^3^Department of Microbiology and Immunology, The Emory Antibiotic Resistance Center, Emory University School of Medicine, Atlanta, GA, United States; ^4^Laboratories of Bacterial Pathogenesis, VA Medical Center, Decatur, GA, United States; ^5^School of Molecular Sciences, University of Western Australia, Crawley, WA, Australia

**Keywords:** anti-virulence, phosphoethanolamine transferase, lipopolysaccharide, multidrug resistance, *N. meningitidis*, *N. gonorrhoeae*

## Abstract

Bacteria cause disease by two general mechanisms: the action of their toxins on host cells and induction of a pro-inflammatory response that can lead to a deleterious cytokine/chemokine response (e.g., the so-called cytokine storm) often seen in bacteremia/septicemia. These major mechanisms may overlap due to the action of surface structures that can have direct and indirect actions on phagocytic or non-phagocytic cells. In this respect, the lipid A (endotoxin) component of lipopolysaccharide (LPS) possessed by Gram-negative bacteria has been the subject of literally thousands of studies over the past century that clearly identified it as a key virulence factor in endotoxic shock. In addition to its capacity to modulate inflammatory responses, endotoxin can also modulate bacterial susceptibility to host antimicrobials, such as the host defense peptides termed cationic antimicrobial peptides. This review concentrates on the phosphoethanolamine (PEA) decoration of lipid A in the pathogenic species of the genus *Neisseria* [*N. gonorrhoeae* and *N. meningitidis*]. PEA decoration of lipid A is mediated by the enzyme EptA (formerly termed LptA) and promotes resistance to innate defense systems, induces the pro-inflammatory response and can influence the *in vivo* fitness of bacteria during infection. These important biological properties have driven efforts dealing with the biochemistry and structural biology of EptA that will facilitate the development of potential inhibitors that block PEA addition to lipid A.

## Introduction

The capacity of *Neisseria gonorrhoeae* (GC) and *N. meningitidis* (MC) to decorate their lipid A with phosphoethanolamine (PEA) has profound implications for their ability to survive host-derived antimicrobials and influence the host’s pro-inflammatory response during infection. In the past decade a number of studies have been reported that advance our knowledge on the molecular mechanisms of this lipid A modification.

We propose that this strategy would render bacteria susceptible to innate host defenses and reduce the potentially damaging action of the pro-inflammatory response during infection. We posit that EptA inhibitors would serve as adjunctive therapeutics to counteract multidrug-resistant strains of *N. gonorrhoeae* (GC) that threaten the efficacy of currently used antibiotics. Accordingly, this review is concerned with bringing together results from molecular and structural studies that have focused attention on the enzyme EptA that is responsible for PEA decoration of lipid A in the context of biological studies that support this modification as a virulence factor.

## The Pathogenic *Neisseria*, Their Diseases and Treatment/Prevention Considerations

*N. gonorrhoeae* causes the sexually transmitted infection termed gonorrhea with a world-wide yearly estimate of >78 million infections (Newman et al., 2015). Gonorrhea is an ancient disease with biblical references (Old Testament; Leviticus 15:1–3). It causes both symptomatic and (frequent) asymptomatic infections at genital and extra-genital sites in both men and women that can have serious consequences for the reproductive and general health of both sexes (summarized in Rice et al., 2017). Symptomatic disease is driven by the pro-inflammatory response and is highlighted by a substantial influx of neutrophils and marked increase in pro-inflammatory chemokines/cytokines. Most often, gonorrhea presents as uncomplicated urethritis in men and cervicitis in women. However, more invasive forms of disease can occur and include epididymitis, pelvic inflammatory disease (endometritis or salpingitis), or disseminated gonococcal infection (DGI) that can involve multiple organs and joints (infectious arthritis) (Rice, 2005). Women suffer the greatest medical complications from invasive GC infections, especially if there is fallopian tube involvement that can result in ectopic pregnancy, and long lasting damage to their reproductive health. Infected mothers can also transmit GC to their newborn during vaginal delivery resulting in ophthalmia neonatorum. Additional extra-genital infections (rectal and oral) in both sexes occur frequently. Finally, repeated GC infections can facilitate transmission or acquisition of the human immunodeficiency virus (HIV) (Malott et al., 2013).

In contrast to GC, MC is frequently carried as a commensal in the nasopharyngeal cavity by a high percentage of the population, but can enter the blood stream and quickly cause life-threatening disease. Invasive meningococcal disease (IMD) syndromes meningitis and/or fulminant septicemia seem to have appeared much later than gonorrhea in the evolution of *Homo sapiens*, with the earliest recorded reports from outbreaks in the early 1800s in Europe and the United States (reviewed in Stephens et al., 2007). Frequently, IMD occurs as localized endemic disease but can also be the cause of larger scale epidemics that include multiple countries and may span continents over decades driven by serial introductions of new variants into susceptible populations by travelers (Yezli et al., 2016). Although sporadic outbreaks of IMD occur throughout Africa, prevalence is highest in the sub-Saharan belt consisting of parts of 26 countries which experiences 7000–180,000 IMD cases annually, typically in a seasonal pattern associated with the dry season (Borrow et al., 2017b). In this region, high temperatures and irritation of mucosal surfaces caused by dust are the most prominent risk factors associated with IMD. While it is not entirely clear why individuals develop IMD, prior events which perturb the mucosal innate immune system (viral infections, smoking, irritants such as dust and dry air) in addition to an increased risk of transmission via respiratory droplets (crowded living, salivary exchange through kissing) increase the probability of contracting IMD. The severity and morbidity of IMD is associated with perturbations in the complement and inflammatory cytokine cascade (Dale and Read, 2013) in addition to immunocompromised states such as asplenectomy (Dionne et al., 2017) and immunotherapy (Winthrop et al., 2018). The risk of repeated episodes of IMD and DGI increase in the absence of an intact functional complement system demonstrating the importance of this arm of the host defence against these infections.

Antibiotic resistance expressed by GC and MC has considerable implications for treatment options affecting severe complications and control of the disease in the community (reviewed in Unemo and Shafer, 2014); this topic, including the biochemistry, genetics and molecular biology of resistance, has been extensively reviewed (Unemo and Shafer, 2014). Briefly, from a historical perspective, beginning with the use of sulfonamides in 1938, which ended in 1942 due to resistance, the efficacy of every antibiotic that has been brought into clinical practice to treat gonorrhea has been removed (penicillin, tetracycline, and fluoroquinolones) or threatened for removal from the treatment regimen due to antimicrobial resistance. The prospect of untreatable gonorrhea due to resistance to approved antibiotics is cause for grave concern (Bolan et al., 2012). While empiric monotherapy was used previously to treat gonorrhea, the continued emergence of multidrug (MDR) resistant strains now requires dual antibiotic therapy consisting of azithromycin and ceftriaxone, but strains resistant to either antibiotic or both have emerged (Ohnishi et al., 2011; Fifer et al., 2016; reviewed in Unemo et al., 2016; Lahra et al., 2018; Public Health England, 2018; Whiley et al., 2018). It is also important to emphasize that the current treatment regimen is costly and not always available in less-resourced settings, which frequently have high incidences of gonorrhea. Clinical trials are in progress with new antibiotics and there is hope that new treatment options will soon be available, however, we should plan for GC to develop resistance to them in due course. In contrast to GC, MC does not appear to be particularly efficient in developing antibiotic resistance or, alternatively, at retaining resistance (a review of MC resistance to antibiotics can be found in Unemo and Shafer, 2014). Although frequently resistant to sulfonamides, MC have remained relatively susceptible to the antibiotics classically used for treatment and chemoprophylaxis but rare instances of penicillinase-producing strains have been reported. MC isolates expressing decreased susceptibility to beta-lactams have emerged during the past two decades and have recently caused an on-going outbreak since 2016 in Australia (Mowlaboccus et al., 2017). Decreased susceptibility and low-level resistance to ciprofloxacin and high-level resistance to rifampin, antibiotics used frequently for chemoprophylaxis to stop the spread of disease during outbreaks, have also been reported from many countries. Thus, while antibiotic resistance in MC is not at the threat level of GC, there is reason for concern that resistance will continue to evolve.

Efficacious vaccines, which are based upon the major capsular serogroups A, C, Y, and W that are responsible for the majority of IMD, have been successful in controlling epidemics (Borrow et al., 2017a). A four-component meningococcal serogroup B (4CMenB) vaccine, Bexsero^®^, has been recommended for pediatric immunization in several countries, including Australia, Canada, United Kingdom, and Italy (Kuhdari et al., 2016) and has shown effectiveness in children as a direct intervention against IMD. Due to the concern regarding emergence of MDR-GC strains and the lack of new antibiotics likely to reach the clinic in the immediate future, a renewed interest in developing a gonorrhea vaccine has developed after years of neglect since the failure of the pilin-based vaccine trials in the early 1980s (reviewed in Jerse and Deal, 2013). Encouragingly, in a population-based survey 4CMenB vaccination was associated with a reduced prevalence of gonorrhea in the vaccinated vs. unvaccinated cohorts in Canada and New Zealand (Petousis-Harris et al., 2017). This has raised the prospect that a vaccine directed toward GC is possible and recent data suggests that the NHBA (neisserial heparin binding antigen) component of 4CMenB raises a sufficiently cross-protective antibody response in mice to be a potential vaccine candidate (Pizza, 2018). In addition, other approaches such as the administration of IL-12 which reverses the immunosuppression by GC during urogenital tract infection is another means of raising a cross-protective antibody response to further infections (Liu et al., 2018). However, the development of a vaccine for GC infection remains elusive as there is no natural correlate of protection such as a protective antibody-response in humans, and further development in this area will need to continue.

## Anti-virulence Strategies Based Upon Models of IMD and Gonorrhea Infection

While approaches to GC vaccination are underway, the prospect of increasing prevalence of MDR-GC is gaining momentum, with the appearance of isolates which are resistant to all classes of routinely used and approved antibiotics for treatment of gonorrhoea. Therefore, the development of novel antimicrobials is an area of intense interest. One approach that has gained momentum in the last decade, is the development of “anti-virulence” compounds which are designed to inhibit virulence thus enabling the natural immune responses of the host to clear the infection (Dickey et al., 2017). To be successful, the virulence target to be inhibited must be essential for the development of disease by the infectious agent, should be tractable to structural studies (crystallography or NMR) and enable high-through put screening strategies to be developed for identifying and optimizing candidate inhibitors.

The model of infection for GC has been excellently summarized by Quillin and Seifert (2018). Upon transmission, GC bind to the epithelial cells of the urogenital tract via type IV pili which retract enabling close contact and the formation of micro-colonies. The micro-colonies release inflammatory mediators: peptidoglycan, lipooligosaccharide (LOS), and outer membrane vesicles which result in the recruitment of neutrophils to the site of inflammation. Since the neutrophils are unable to clear the infection, the influx of neutrophils form a purulent exudate that then facilitates transmission to the urogenital tract of the next partner. *N. gonorrhoeae* can also colonize the nasopharynx and until recently this was considered transient and not a significant mode of transmission. However, antibiotic treatment failure is most commonly associated with nasopharyngeal carriage and often necessitates a nasopharyngeal swab test to ensure total cure after therapy (Unemo et al., 2016).

*Neisseria meningitidis* is most commonly carried asymptomatically in the nasopharynx of 10% of young adults. It is transmitted via the respiratory route in salivary droplets (Stephens et al., 2007). The MC model of colonization of the nasopharynx also involves type IV pili to initiate attachment and then close adhesion to the host epithelium when retracted. However, the meningococcal model of invasion also includes a wider variety of adhesins required for interaction with endothelial cells lining the blood vessels during IMD (Stephens et al., 2007).

Interestingly a single virulence factor, the ethanolamine transferase EptA (formerly termed lipid A phosphoethanolamine transferase LptA), has been shown to be required for multiple aspects of GC and MC pathogenesis including colonization, inflammation and survival in neutrophils, and we propose this enzyme has exciting potential as a target for development of anti-virulence compounds.

## Function Of EptA

**FIGURE 1:**
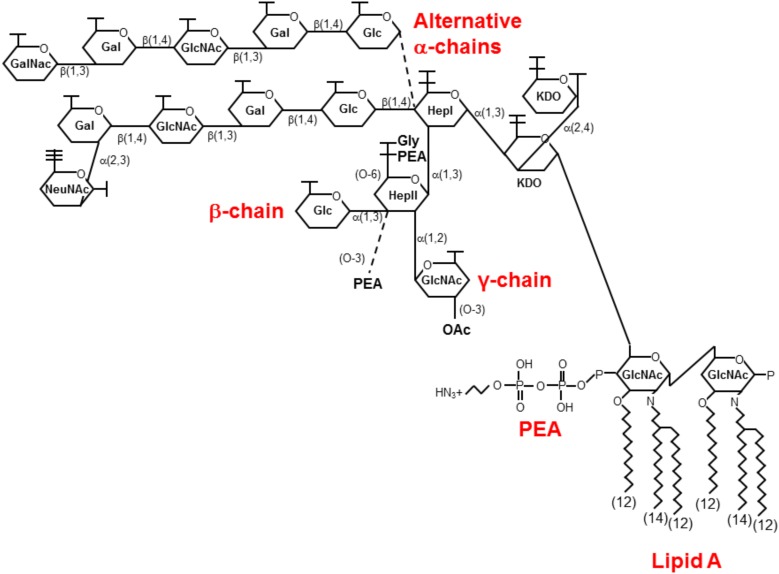
The structure of LOS from pathogenic *Neisseria* spp. Shown is a predominant LOS isoform produced by the pathogenic *Neisseria*. The glycosidic α, β, and γ chains are attached to a conserved core of heptose (Hep) and keto-octulosonic residues (KDO) attached to lipid A. This figure shows two alternative a-chains commonly expressed by *N. gonorrhoeae* terminating with GalNAc and *N. meningitidis* terminating in NeuNAc. Variable glycoforms are expressed upon the phase variable expression of various glycosyltransferases (reviewed in Bartley and Kahler, 2014). PEA is shown attached to the 4′ position of lipid A in this representation, but variable phosphoforms have been identified with substitutions also occurring on the 1 position (see text and Lewis et al., 2009; John et al., 2012). Gal, galactose; Glc, glucose; GlcNAc, *N*-acetyl-glucosamine; NeuNAc, N-acetyl-neuraminic acid; P, phosphate; PEA, phosphoethanolamine; OAc, O-acetyl group.

EptA is an enzyme required for the decoration of lipid A of the LOS of the outer leaflet of the outer membrane of *Neisseria* spp. (Cox et al., 2003a). The LOS structure of the pathogenic *Neisseria* spp., is identical being composed of a conserved inner core consisting of heptose (Hep) and 3-*deoxy*-D-*manno*-2-octulosonic acid (KDO) attached to a lipid A moiety embedded in the outer membrane (**Figure 1**; Bartley and Kahler, 2014). Substitutions to this inner core are variable and contribute to the distinct immunoreactivity profiles of these structures. All structures have an α-chain, β-chain, and γ-chain attached to the heptose residues, HepI and HepII, of the conserved inner core. The α-chain attached to HepI is composed of either a lacto-*N*-neotetraose (LNT, Galβ1, 4GlcNAcβ1, 3Galβ1, 4Glc where Gal is galactose, GlcNAc is *N*-acetyl-glucosamine and Glc is glucose) which mimics human glycosphingolipids such as the human I erythrocyte antigen (Mandrell et al., 1988) or a di-galactose (Galα1, 4Gal) which mimics the human P^k^ antigen (Mandrell and Apicella, 1993). The β-chain attached to HepII is also variable consisting of either O-3 linked PEA groups (Mackinnon et al., 2002), or a O-3 linked glucose (Banerjee et al., 1998) attached to HepII of the conserved inner core. Lastly the γ-chain attached to HepII is conserved with the addition of a α-2 linked *N*-acetyl-glucosamine (GlcNAc) residue (Kahler et al., 1996) which is variably substituted with an O-acetyl group at position 3 or 6 (Kahler et al., 2006; Bartley and Kahler, 2014).

The lipid A structure is conserved within the *Neisseria* spp. (Johnson et al., 1975; John et al., 2012). All species produce a major hexaacyl lipid A with the hydroxyl groups at positions 3 and 3′ carrying (*R*)-3-hydroxydodecanoic acid [12:0 (3-OH)] and the amino groups at positions 2 and 2′ being substituted with (*R*)-3-(dodecanoyloxy)tetradecanoic acid [3-O (12:0)-14:0]. The β1-6 linked di-glucosamine backbone of lipid A can be variably substituted at the 1 and 4′ positions with phosphate, di-phosphate, *O*-phosphorylethanolamine, and *O*-pyro-phosphorylethanolamine (Zughaier et al., 2007). This remarkable heterogeneity appears to be characteristic of GC, MC, *N. lactamica*, and some *N. polysacchareae* but not other commensal *Neisseria* spp. (John et al., 2012).

**FIGURE 2:**
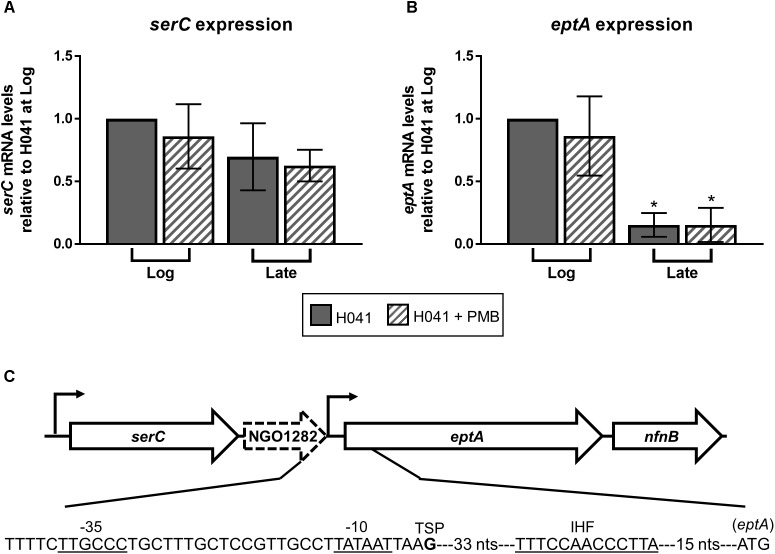
Expression of *eptA* is growth phase-dependent. qRT-PCR analysis of **(A)** *serC* and **(B)** *eptA* expression in GC isolate H041 grown in GC broth with and without the addition of 1 μg/ml polymyxin B (PMB). RNA samples were collected during logarithmic growth (Log, OD_600_ of ∼0.4) and at the end of logarithmic growth (Late, OD_600_ of ∼1.0) and subject to qRT-PCR using gene-specific primers. Primers serC_qRT_F (5′-TGTTGCCTGAAGCTGTGTTG-3′) and serC_qRT_R (5′-TGTTCCGCATGATGCAGGAT-3′) were used for *serC* expression. Primers lptA_qRT_F (5′-GGCATCGCGATGTTGCAATA-3′) and lptA_qRT_R (5′-CACGACCGCCATATCCAATTG-3′) were used for *eptA* expression. 16S rRNA expression was assessed with primers 16Smai_qRTF (5′-CCATCGGTATTCCTCCACATCTCT-3′) and 16Smai_qRTR (5′-CGTAGGGTGCGAGCGTTAATC-3′). The means and standard deviations of three biological replicates are shown. The expression of *serC* and *eptA* were normalized to 16S rRNA expression at the Log timepoint without PMB treatment. Gene expression data were analyzed by ANOVA followed by Sidak’s multiple comparisons test. ^∗^Indicates *P* ≤ 0.05. **(C)** Organization of *eptA* locus and position of promoters modified from Kandler et al. (2014). The *eptA* -10 and -35 promoter elements, transcriptional start point (TSP) is the bolded G nucleotide, putative integration host factor binding site (IHF) based on the consensus IHF-binding site in *E. coli* (11 matches of 13 bp) previously described in *N. gonorrhoeae* (Lee et al., 2006) as indicated by the underlined sequence, and the translational start codon are shown.

The lipid A phosphoforms in MC and GC are produced by EptA which is a cytoplasmic-membrane bound enzyme facing the periplasm encoded by a gene found in the pathogenic *Neisseria* but absent in most commensal species (Cox et al., 2003a; John et al., 2012). Once the LOS structure is processively synthesized in the cytoplasm (Bartley and Kahler, 2014) it is transported through the periplasm to the outer membrane by the ABC transporter complex consisting of lipopolysaccharide transport proteins (Lpt), LptBCFG. The lipopolysaccharide chaperone LptA transfers the LOS to the LptED complex in the outer membrane which inserts the LOS into the outer leaflet of the outer membrane (Bos et al., 2004). Only a proportion of lipid A is decorated with PEA headgroups and this can vary between strains by up to 60% of lipid A molecules (Liu M. et al., 2010; Liu X. et al., 2010; Piek et al., 2014; John et al., 2016). A number of transcriptional and post-translational mechanisms controlling EptA expression have been identified that collectively result in strain variation of lipid A phosphoforms. *eptA* is co-transcribed in an operon with *serC*, which encodes a putative phosphoserine aminotransferase, a hypothetical gene (NGO1282), and *nfnB*, which encodes a putative nitroreductase [**Figure 2** below; (Kandler et al., 2014)]. Expression of *eptA*, but not *serC*, is growth phase-dependent with maximal expression observed at the mid-logarithmic phase in GC. It is not yet clear why transcription of *eptA* is maximal during exponential growth, but the presence of a putative integration host factor (IHF) binding site 37 bp downstream of -10 hexamer of the proximal *eptA* promoter might provide a mechanism (see **Figure 2C** for region of similarity with IHF-binding sites). Previous work by Hill et al. (1998) has shown that mRNA of IHF declines as GC enters stationary phase consistent with a potential means of influencing transcription in a growth phase dependent manner.

EptA expression is governed at the translational level by two means: phase variation of the open reading frame and protein stabilization by oxidoreductases. Translation of full-length EptA is subject to high frequency mutation due to a phase variable poly-T track in the 5′ end coding sequence (Kandler et al., 2014). In the “phase-on” position, the tract consists of 8 Ts. At a frequency of 10^-4^ (4 logs greater than spontaneous mutation) a single T insertion occurs due to slipped-strand mispairing resulting in premature truncation of EptA (62 amino acid protein).

The second mechanism of post-translation control of EptA expression is a result of the requirement for disulfide bonds that improve stability of the protein in the periplasm. Five disulphide bonds are donated by oxidoreductases to EptA as it is transported via the Sec pathway into the periplasmic space. *N. gonorrhoeae* and MC contain a cohort of three oxidoreductases (DsbA1, DsbA2, and DsbA3) which are oxidized at the active site with a disulphide bond by membrane bound DsbB. The oxidoreductase rapidly and irreversibly donates the disulphide bond to thiol groups on substrate proteins resulting in the release of an oxidized substrate carrying a disulphide bond and a reduced oxidoreductase. DsbA1 and DsbA2 are paralogs, with DsbA2 being encoded on a genetic island that is distributed in MC but absent in GC (Perrin et al., 2002). Both DsbA1 and DsbA2 are bound to the cytoplasmic membrane by a lipid-linked anchor (Tinsley et al., 2004) and have an overlapping repertoire of substrates involved in pilin biogenesis (Tinsley et al., 2004; Sinha et al., 2008). In contrast, DsbA3 is a soluble periplasmic enzyme, has a stronger oxidizing potential than that of the other DsbA enzymes and has no involvement with pilin biogenesis (Vivian et al., 2008, 2009). All three oxidoreductases have the capacity to introduce disulphide bonds into EptA, however, only the loss of DsbA3 resulted in protein instability and loss of function (Piek et al., 2014). Currently it is not clear whether this post-translational modification pathway is regulated as no change in expression of oxidoreductases has been detected in the many studies to-date on regulatory pathways in MC or GC (summarized in Piek and Kahler, 2012).

## Structure And Functional Model Of EptA

**FIGURE 3:**
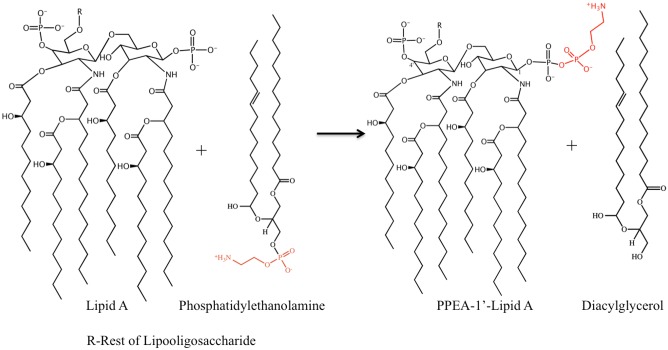
Reaction catalyzed by EptA. EptA is an esterase that cleaves PEA from phosphatidylethanolamine to release di-acylglycerol. The PEA remains transiently attached to the active site Thr280 residue before being transferred to the lipid A head group. PEA has only been transferred to the 1 position of lipid A in this figure however the 4′ position can also accommodate a PEA moiety.

**FIGURE 4:**
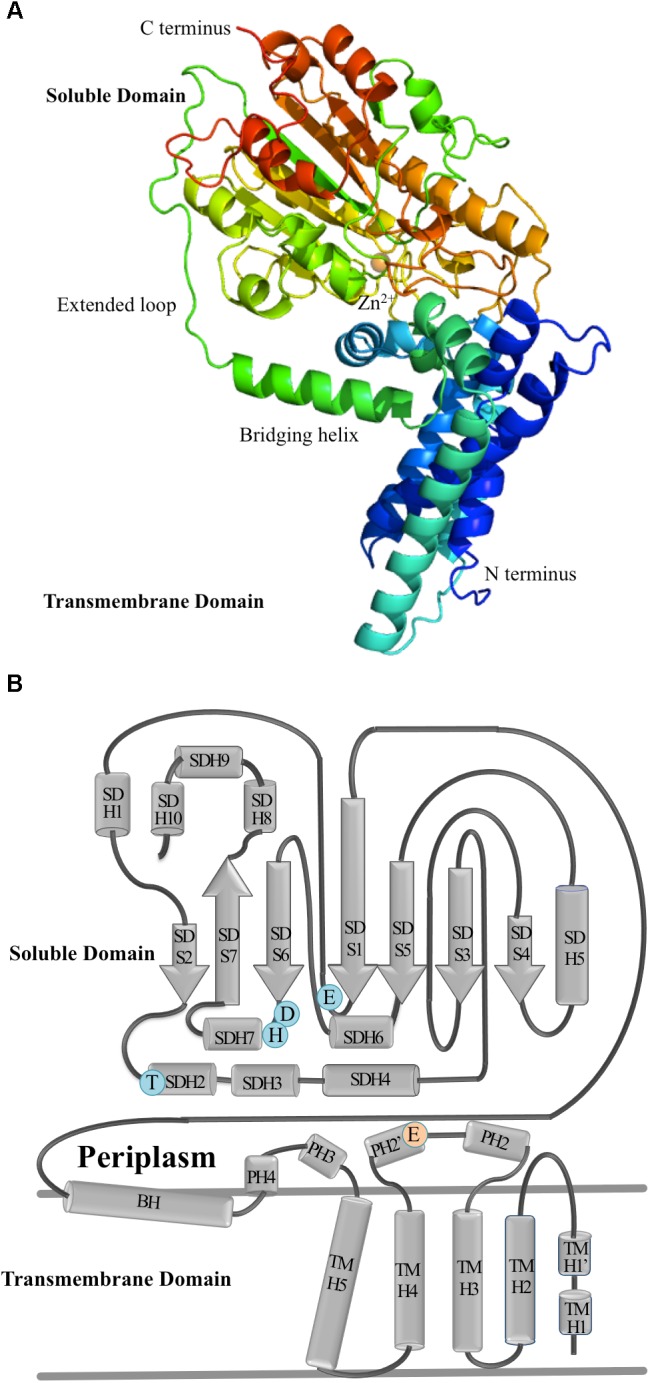
The molecular structure of EptA. **(A)** A ribbon diagram showing the secondary structure elements of EptA. Coloring from blue to red corresponds to the N- and C- termini, respectively. The orange sphere denotes the position of the Zn^2+^ ion. **(B)** Topology diagram of EptA. The cylinders correspond to helices and the arrows correspond to the strands. Key amino acid residues making up the active site of the enzyme are shown in blue and orange circles. Blue circles correspond to residues which coordinate the Zn^2+^ ion and the orange circle corresponds to a conserved glutamate residue proposed to interact with the amine group of PEA.

EptA catalyzes the transfer of PEA from phosphatidylethanolamine (PtdE) to lipid A at 1 and/or 4′ head group positions (**Figure 3**). EptA is an integral membrane protein consisting of an N-terminal transmembrane (TM) domain and a C-terminal soluble periplasmic-facing domain. The solved crystal structure of the soluble domain showed it adopts a hydrolase-type fold with a bound Zn^2+^ ion at the enzyme active site near to the catalytic nucleophile, Thr280 (Anandan et al., 2012; Wanty et al., 2013). The soluble domain, although retaining the esterase activity required for cleavage of PtdE, was inactive as a lipid A transferase in bacterial cells suggesting that the TM domain is important for overall function. The solved 3D structure of full-length EptA revealed that the N-terminal TM domain was connected to the soluble domain by a bridging helix and an extended loop (Anandan et al., 2017; **Figure 4**).

The membrane domain contains five transmembrane helices (TMH1-5, **Figure 4**) oriented approximately parallel to one another and spanning the inner membrane in a previously uncharacterized fold. A patch of positively charged residues (Lys142, Lys144, Arg146, and Lys150) at the cytoplasmic end of TMH5 are likely to provide interactions with the phospholipid head groups at the inner membrane surface. The bridging helix (residues 194–208) has a highly unusual configuration in the structure and is linked to the soluble domain by an extended coiled region (residues 210–231). There is a large interface between the soluble and membrane domains which is conserved in all lipid A PEA transferases suggesting this region has a conserved function. Intrinsic tryptophan fluorescence experiments and molecular dynamics simulations has led to the proposal that the protein is highly dynamic in the membrane and support a model that the enzyme carries out catalysis through a “ping pong” kinetic mechanism. In this model, the first step requires EptA to bind PtdE using the small periplasmic helices (PH2 and PH2′) located between TM3 and TMH4. PEA is cleaved from PtdE and transferred to the Thr280 residue to form an PEA-enzyme intermediate. The soluble domain then rotates way from the TM domain, resulting in an open conformation capable of accepting the larger lipid A into the binding site pocket and completing the transfer of the PEA from the enzyme intermediate to the 1 and 4′ positions of lipid A.

## Role of EptA in Colonization of Mucosal Surfaces

*N. meningitidis* and *N. gonorrhoeae* display tissue tropism toward nasopharyngeal or urogenital mucosal surfaces, respectively, and models of infection have concentrated on identifying colonization factors needed for these sites (Stephens et al., 2007; Rice et al., 2017). EptA mutants of both organisms are less successful at colonization in these models. EptA mutants of MC colonize epithelial cell monolayers at lower rates than the wild-type control strain (Takahashi et al., 2008). Removal of PEA groups on lipid A leaves free phosphates which at neutral pH will be negatively charged. In theory, the increased negative charge of the bacterial surface increases repulsion forces between the bacterial cell surface and the host cell disrupting the initial colonization interactions of the bacterial adhesins with host cell receptors. A second possibility is that the change to lipid A structure in addition to surface charge may also affect the positioning and function of outer membrane adhesins thus affecting attachment and invasion (Tommassen and Arenas, 2017). Although this effect on attachment has been observed in cell monolayer models of infection for both MC and GC, *eptA* mutants of GC were as efficient as wild-type strains in colonizing the urogenital tract of the female mouse model (Packiam et al., 2014). No study has as yet examined the significance of *eptA* “phase off” mutants of GC or MC in clinical infections of humans. Diagnosis by culture requires selective culture of clinical samples on modified Thayer Martin agar which contains 10 μg/ml of colistin (polymyxin E) that suppresses the growth of most Gram-negative bacteria but not *Neisseria* spp. which are resistant to this antibiotic. This unfortunately prevents the detection of MC or GC with “phase-off” *eptA* in pathology diagnostic laboratories as “phase-on” *eptA* confers colistin resistance.

## Role Of EptA in Resistance to Complement Mediated Killing By Normal Human Serum

The complement pathway is an important arm of the innate immune system. Complement protects against infection and under physiological conditions, its activation is tightly controlled by soluble and membrane-associated complement inhibitors. Both GC and MC have sophisticated mechanisms for evading complement-mediated killing during colonization and invasive disease (Ram et al., 1999, 2016). The main mechanism of resistance to complement-mediated killing in MC is determined by the polysaccharide capsule which inhibits the insertion of the membrane attack complex (MAC). An intact LOS structure is also required for full resistance to complement but lipid A decoration with PEA appears to have no discernible effect (Cox et al., 2003b; Pizza and Rappuoli, 2015). In contrast, in GC which lacks a polysaccharide capsule, the loss of PEA decorations on the lipid A results in susceptibility to complement-mediated lysis. PEA on the lipid A headgroups increases the binding of the complement regulatory protein C4b binding protein (C4BP) to porin B (PorB), thus preventing the activation of the classical complement pathway (Lewis et al., 2009, 2013). Loss of PEA from lipid A also affected binding of the alternative pathway regulator, factor H (fH), to PorB of some strains (Lewis et al., 2013). Altogether lipid A decorated with PEA alters binding of C4BP and fH to PorB and contributes to the ability of GC to resist complement-mediated killing by both the classical and alternative complement pathways.

Evasion of complement-mediated killing is commonly associated with escaping the ability of normal human serum to the kill the organism once it enters the bloodstream. In this case, the role of complement resistance by MC during invasive disease is substantial in enabling bacteremia (Lewis and Ram, 2014). Female and male genital tract secretions have high levels of immunoglobulin IgG and complement factors (Birse et al., 2013; Horton and Vidarsson, 2013). In the model of cervicitis proposed by Edwards (2008), the lack of a detectable antibody response in uncomplicated gonococcal cervicitis means there is an absence of immunoglobulin to initiate the classical complement pathway. However, the binding of fH to the bacterial surface by PorB results in rapid inactivation of complement factor C3b to iC3b, which forms bridges with the host cell receptor CR3 (complement-receptor 3). CR-3 mediates endocytosis of the bacteria into the cervical epithelia potentially leading to the ability to colonize the mucosal surface without triggering an inflammatory response, a characteristic of the asymptomatic phase of gonorrhea seen in both sexes. Therefore, the interplay of complement-regulatory proteins on the gonococcal cell surface is closely associated with colonization events in men and women (Edwards and Apicella, 2004).

## Role of EptA in the Stimulation of the Pro-Inflammatory Responses of Macrophages and Neutrophils

While the endotoxins of MC and GC have an identical lipid A, most information on the structure and functional relationships that are necessary for eliciting the pro-inflammatory response has been conducted with meningococcal lipid A (Zughaier et al., 2005, 2007; Zimmer et al., 2007). Both endotoxins induce pro-inflammatory responses from cells via the Toll-like receptor (TLR) 4/myeloid differentiation factor 2 (MD-2) complex which sits in the host cell membrane and receives endotoxins from the soluble CD14 protein which binds endotoxins in serum (Park et al., 2009). Activation of TLR4/MD2 is dependent upon the conformational structural variations in lipid A, degree of lipid A phosphorylation, number and length of acyl chains, net charge of the molecule, and variations in hydrophobicity (Zimmer et al., 2007). The role of lipid A decorated with PEA headgroups in eliciting a stronger cytokine response was subsequently confirmed by John et al. (2012) and Liu M. et al. (2010) using purified lipid A with and without PEA decorations to elicit cytokine responses from cell monolayers. Packiam et al. (2014) further showed that purified LOS containing lipid A devoid of the PEA modification from a GC *eptA* mutant induced significantly lower levels of NF-κB in human embryonic kidney Toll-like receptor 4 (TLR4) cells and murine embryonic fibroblasts than wild-type LOS of the parent strain. Consistent with these tests, analysis of the chemokine/cytokine responses of female mice with lower genital tract infection showed that the parent GC strain possessing lipid A with PEA decorations elicited a robust pro-inflammatory response that was significantly dampened in mice infected with the GC *eptA* null mutant; while markers of the anti-inflammatory response were not significantly different in mice infected with either strain.

The binding of lipid A to the TLR4/MD-2 complex on macrophages and neutrophils results in cellular activation, the release of cytokines, chemokines, reactive oxygen species (ROS), and nitric oxide which will kill phagocytosed bacteria. In the female mouse model of lower genital tract infection, Packiam et al. (2014) reported that the GC *eptA* mutant had a substantial fitness defect *in vivo* compared to the wild-type strain during a competitive, mixed infection. Hobbs et al. (2013) made similar observations in human male volunteers. They suggested that the observed fitness defect in the competitive infection model was due to the strong pro-inflammatory response elicited by the wild-type GC strain that was sufficient to activate macrophages and neutrophils which effectively killed the GC *etpA* mutants.

Macrophages and neutrophils kill bacteria by phagocytosis followed by the formation of a phagolysosome which utilizes oxidative mechanisms (ROS such as hydrogen peroxide, superoxide etc.) and non-oxidative mechanisms [serine proteases, cationic antimicrobial peptides (CAMPs), and iron sequestration] to kill the bacteria (Criss, 2014). Neutrophils will also secrete neutrophil extracellular traps (NETs) consisting of DNA coated with CAMPs such as LL-37 to trap the bacteria and kill them. Wild-type MC and GC evade killing by neutrophils by avoiding phagocytosis, engaging surface regulatory proteins that trigger an oxidative burst before the bacteria are phagocytosed, and using mechanisms that quench or detoxify ROS and resist CAMPs (Criss, 2014). The *eptA* mutants from MC and GC are exquisitely sensitive to killing by CAMPs and are rapidly killed by neutrophils as a result (Tzeng et al., 2005; Kandler et al., 2014; Handing and Criss, 2015; Tzeng and Stephens, 2015). Interestingly, decoration of lipid A with PEA has also been shown to interfere with the maturation of the phagolysosome by delaying fusion of azurophilic granules with maturing phagolysosomes (Johnson and Criss, 2013; Handing and Criss, 2015) and additionally dysregulates autophagy, a process which normally targets surface macromolecules including bacteria, to the phagolysosome (Zughaier et al., 2015).

## Conclusion

The decoration of lipid A with PEA in GC and MC is an essential pathogenesis factor that distinguishes these pathogens from most commensal *Neisseria* spp. This feature stimulates the pro-inflammatory responses during IMD and gonorrhea but also provides a protective role against clearance by innate immune cells such as neutrophils and macrophages that are attracted to the site of infection. In both instances, the engulfment of the bacteria leads to a frustrated innate immune response that results in chronic inflammation and in the case of GC infected neutrophils become vehicles for transmission between hosts.

We propose that inhibition of EptA will improve killing and clearance of these pathogens by neutrophils thus improving clearance of infection from mucosal surfaces and providing a mechanism that curtails transmission of GC in neutrophils. In addition, EptA enzymes are found in many gram-negative pathogens (e.g., *Escherichia coli*, *Salmonella enterica*, *Klebsiella pneumoniae*, etc.), rendering them resistant to colistin therapy (Gao et al., 2016). Detailed studies are underway to identify and optimize potential EptA-inhibitors that suppress expression (Daly et al., 2017) or inhibit the enzyme directly in these bacterial species. We suggest similar approaches to inhibition of EptA from *Neisseria* sp. will be prove to be a beneficial approach to the development of novel therapies. Therapeutics to boost the bactericidal activity of phagocytic cells are currently in development (Munguia and Nizet, 2017), which in combination with anti-EptA compounds, could be used as novel combination therapies to effectively reduce transmission of multi-drug resistant GC and MC isolates.

## Author Contributions

CK and WS wrote the drafts of the manuscript with editorial changes and input by KN, AA, and AV. AA and KN prepared the figures and performed experiments described in the text.

## Conflict of Interest Statement

The authors declare that the research was conducted in the absence of any commercial or financial relationships that could be construed as a potential conflict of interest.
